# Identifying Potential Sources of Exposure Along the Child Feces Management Pathway: A Cross-Sectional Study Among Urban Slums in Odisha, India

**DOI:** 10.4269/ajtmh.16-0688

**Published:** 2017-07-17

**Authors:** Fiona Majorin, Belen Torondel, Parimita Routray, Manaswini Rout, Thomas Clasen

**Affiliations:** 1 Department of Disease Control, Faculty of Infectious and Tropical Diseases, London School of Hygiene and Tropical Medicine, London, United Kingdom;; 2 Department of Environmental Health, Rollins School of Public Health, Emory University, Atlanta, Georgia

## Abstract

Child feces represent a particular health risk to children due to increased prevalence of enteric agents and a higher risk of exposure owing to exploratory behaviors of young children. The safe management of such feces presents a significant challenge, not only for the 2.4 billion who lack access to improved sanitation, but also due to unhygienic feces collection and disposal and poor subsequent handwashing practices. We assessed potential sources of fecal exposure by documenting child feces management practices in a cross-sectional study of 851 children < 5 years of age from 694 households in 42 slums in two cities in Odisha, India. No preambulatory children and only 27.4% of ambulatory children defecated directly in the latrine. Children that did not defecate in a latrine mainly defecated on the ground, whether they were preambulatory or ambulatory. Use of diapers (1.2%) or potties (2.8%) was low. If the feces were removed from the ground, the defecation area was usually cleaned, if at all, only with water. Most children’s feces were disposed of in surrounding environment, with only 6.5% deposited into any kind of latrine, including unimproved. Handwashing with soap of the caregiver after child feces disposal and child anal cleaning with soap after defecation was also uncommon. While proper disposal of child feces in an improved latrine still represents a major challenge, control of the risks presented requires attention to the full range of exposures associated to the management of child feces, and not simply the place of disposal.

## INTRODUCTION

Worldwide 2.4 billion people did not have access to improved sanitation in 2015, including nearly 1 billion people that practiced open defecation.^1^ India represents a particular challenge, as 44% of its population practiced open defecation and only 40% used improved facilities.^1^

Poor sanitation is a major cause of fecal–oral diseases, including diarrhea which is responsible for more than 1.2 million deaths annually.^2^ Several systematic reviews have linked improved sanitation with lower risks of diarrhea,^3–8^ soil-transmitted helminth infections,^3,9,10^ schistosomiasis,^3,11^ and trachoma.^12,13^ Unsafe sanitation (access to unimproved facilities or improved without a sewer) was estimated to be responsible for 808,000 deaths (all causes) and 46,275,000 DALYs in 2015, mostly due to diarrhea.^14^

The unsafe disposal of child feces represents a particular challenge for preventing transmission of fecal–oral diseases, particularly among young children. First, young children have the highest incidence of enteric infections^15^ and their feces are most likely to contain transmissible pathogens.^16^ Second, young children tend to defecate in areas where other susceptible children could be exposed.^17^ This exposure is worse for young children due to their higher vulnerability which is a function of the time they spend on the ground and exploratory behaviors including geophagia,^18,19^ which has been associated with enteropathy,^20^ as well as their immature immune system.^21^ Third, diarrhea is one of the main causes of death of young children making them most vulnerable to fecal exposure.^2^

A recent systematic review suggests that safe disposal of child feces may also play a role in preventing diarrhea (F. Majorin, submitted). However, most of the evidence was judged to be of low quality using GRADE criteria^22^ and no trials that measured health outcomes focused on improving child feces disposal only. Another recent systematic review also found a lack of evidence on the effectiveness of interventions to improve child feces disposal.^23^ Recent evidence from a cohort study in rural Bangladesh found that children from households that disposed of their children’s feces unsafely had higher scores of enteropathy, a disorder of the small intestine which is thought to lead to undernutrition and growth faltering, and greater odds of being wasted.^24^ The same study found that households that practiced unsafe child feces disposal had more than five times greater odds of having pathogenic *Escherichia coli* in the soil of the areas where study children played compared with households that practiced safe child feces disposal, supporting the hypothesis that unsafe child feces disposal may increase the risk of exposure to enteric pathogens.^24^

Even in settings with improved sanitation (or “basic sanitation” under the proposed SDG sanitation ladder^25^), householders often do not dispose of child feces in latrines.^26,27^ A recent report by the World Bank Water and Sanitation Program (WSP) presenting analysis from the latest available Multiple Indicator Cluster surveys (MICS) and Demographic and Health Surveys (DHS) (survey years: 2006–2012) found that in 15 of 26 locations more than 50% of households reported disposing of their youngest under 3-year-old child’s feces unsafely (not into a latrine); and the percentage of feces ending up in improved sanitation facilities was even lower.^28^ In India, the latest DHS (2005–2006) found that only 20.3% of child feces ended up in a latrine (child defecated in latrine [11.5%] and 8.8% were disposed in the latrine), and 0.8% was buried.^29^ A cross-sectional study of child feces disposal practices among rural households in villages in the State of Odisha, India, where the Total Sanitation Campaign (TSC) had been implemented at least 3 years before, found that 81.4% of child feces were disposed of unsafely, with the majority of feces reported being deposited with the solid waste. Safe disposal of child feces only occurred in households with latrines, but the majority of the feces were disposed of elsewhere.^27^

While the Government of India has endeavored to reduce open defecation and improve sanitation through a series of initiatives, studies have reported no significant impact of the interventions on diarrhea, soil-transmitted helminth infection, or nutrition.^30,31^ In one such evaluation, the intervention increased the safe disposal of child feces from 1.1% at baseline to 10.4% in intervention households compared with 3.1% in the control households (RR: 3.34; 95% CI: 1.99–5.59).^26^ However, this increase in safe child feces disposal was directly related to increases in latrine coverage in the intervention communities and not from a change in underlying behaviors.

We undertook this cross-sectional study to describe the child feces management practices of children under 5 years of age in urban slums in Odisha, India. The study is a complement to our previous work in rural villages in Odisha.^27^ While the DHS and MICS surveys collect limited data on child feces disposal behaviors, such surveys do not always cover informal settlements such as urban slums.^1^ In addition, since they only have one question on child feces disposal practices (“The last time [name of youngest child] passed stools, what was done to dispose of the stools?”),^32^ they do not describe the range of child feces management behaviors. We sought to describe a more comprehensive range of intermediary behaviors that may cause exposure to child feces, including where the child defecates, where the feces are disposed of, and any associated hygiene behaviors.

## MATERIALS AND METHODS

### Study design and setting.

The study followed a cross-sectional design using a questionnaire, spot checks, and demonstrations of child feces management practices as data collection tools. The data collection took place in July and August 2014.

### Slum selection.

The informal settlements (slums) were selected from lists of 23 slums in Cuttack and 39 slums in Bhubaneswar in which other sanitation-related studies had been conducted.^33,34^ The selection criteria for the slums was that they had at least 33 households with access to either individual household latrines or functional community latrines.^33,34^ We excluded three leprosy colonies from our list of eligible slums as well as slums in which pilot activities were previously conducted. This selection process resulted in 20 eligible slums in Cuttack and 28 eligible slums in Bhubaneswar. These slums were randomly ordered for each city using STATA version 12 (StataCorp, College Station, TX).

### Sample size calculation.

The primary outcome for this cross-sectional study is the proportion of children < 5 years of age whose feces are disposed of safely (defined here as defecation or disposal in a latrine). Based on previous studies, the sample size was calculated using the average of 30% safe disposal. Using simple random sampling, the average of 30% safe disposal of child feces led to a sample size for frequency in a population of 323 people (95% confidence).^35^ The sample size calculation was adjusted to account for clustering, with an intracluster correlation coefficient (ICC) of 0.06 based on previous work in rural Odisha.^30^ Based on the different sample size calculations in different scenarios, 20 households in 35 clusters (a total of 700 households) was chosen to be the best logistical option. The study was not separately powered for each city but for 35 slums in total. As it was not always possible to find 20 eligible households in each selected slum, we continued selecting slums in the order in which they had been randomly ordered until we reached our target sample size of 700 households. This resulted in the data being collected in 42 slums: 22 in Bhubaneswar and 20 in Cuttack.

### Household selection.

In the selected slums, households eligible for inclusion in the study were required to meet the following eligibility criteria: 1) have at least one child < 5 years of age with a primary caregiver older than 18 years, and 2) the primary caregiver reported having access to sanitation facilities (individual household latrines, shared or communal facilities^34^) or belonged to a slum with communal sanitation facilities. For this purpose, shared latrines are facilities used by more than one household, including neighbors, families, or tenants/landlords. Communal latrines are facilities managed by the community or pay-per-use facilities run by a third party. Households that otherwise met such eligibility criteria were nevertheless excluded from the study if the primary caregiver was an ASHA (Accredited Social Health Activist), *anganwadi* (government sponsored child-care and mother-care center) worker, or a person who had worked for health promotion campaigns.

As a sampling frame, we initially envisaged using lists of < 5-year-old children managed by *anganwadi* workers in their respective slums. This method was not feasible due to issues with finding the randomly selected households in the slum. Instead participating households were selected through systematic sampling using an adaptation of the Extended Program of Immunization (EPI) sampling method.^36^ This method involved the supervisor spinning a pen in a central location of the slum to determine the direction in which each enumerator would select households. The four enumerators enrolled every other household on the left that fit the eligibility criteria in that direction until they each had collected data from five households, the slum boundary was reached or it was the end of the field day. When the enumerators reached the slum boundary before having collected data from five households, they would go back to the last intersection or the central point (if no intersections were met) and start the process again.

The number of participating households in each slum varied due to the varying sizes of the slums and the availability of households with children < 5 years of age at the time of visit. Respondents were the primary caregivers (defined as “the one who usually cares for the child”) of the youngest child < 5 years of age in each household. Households that were locked, where the primary caregiver was unavailable at the time of visit, that did not meet the eligibility criteria, or that refused to participate, were not enrolled and the enumerators would go to the next household on the left until they found one that met the eligibility criteria.

### Data collection tools.

Data collection tools included a structured survey, which included questions on socioeconomic and demographic factors, access to sanitation, water and hygiene facilities, availability of potties and diapers, exposure to messages about child sanitation or hygiene, and agree or disagree statements. Questions about defecation place and feces disposal method for the last time each child < 5 years defecated^27^ were included using wording as per the core questions of the WHO/UNICEF Joint Monitoring Program on Water and Sanitation (JMP).^32^ Questions were also asked to know “on what” the child defecated (if directly on the ground or on paper or polythene, etc.) and “what” was used to dispose of the stools. The age and mobility capacity (whether the child can or cannot walk) of the children, whether they were exclusively breastfed and the consistency of their feces (solid, liquid, semisolid) the last time they defecated, were also recorded. Data were also collected on the age, marital status, and usual defecation places of each family member over the age of 5 years.^37^

Spot checks were done to determine the type of the latrine (flush/pour flush with pit/closed sewer system, flush/pour flush without pit/open sewer system, pit latrine with slab, or other), reported by the households as the one used the majority of the time and whether it looked used (if there was smell or the pan was wet or there were stains of urine/feces),^30^ to check the presence of a potty in the household, whether children were wearing a diaper, and to check the availability of soap and water at the specific place identified by participants to be used for handwashing after disposal of child feces. The primary caregiver was also asked to demonstrate (using plastic feces) how he/she would manage the stool if the youngest child defecated at the time of visit. The enumerators would prompt the caregiver to explain and/or show all the steps from the moment the child defecated.

The questions on defecation and disposal practices for the last time the children defecated were asked for all the children < 5 years in each household (defined as sharing the same cooking pot). As such, data could be collected on children that were cousins or siblings, as long as they lived in the same house and the parents shared the same cooking pot.

The disposal sites/places were recorded so that the place where most feces ended up was recorded, for example, if the child defecated in his pants and the pants were washed in water, the disposal site was recorded as washed with water. If on the other hand the caregiver first put the feces in the latrine or garbage and then washed the pants, which might have contained some remains of feces, the disposal site was recorded as latrine or garbage.

The questionnaire, information sheet, and consent forms were written in English and then translated into Odia, the local language. A researcher bilingual in Odia and English evaluated the translations. All the enumerators who conducted the surveys were fluent Odia speakers. During the development of the questionnaire, the field supervisors piloted the questions in a slum and the questions were amended accordingly.

### Field procedures.

After training and piloting in two slums with retraining after the first pilot, the field team collected data from two slums per day (one in Cuttack and one in Bhubaneswar) as far as possible and weather permitting (data collection occurred during the monsoon season). The field team was divided into two teams of four female enumerators and supervised by one or two field supervisors (two males and one female) depending on the size of the slum that was visited. When the team arrived at a slum they would start by visiting the community latrine(s) if there were one or several, where they would conduct spot checks of the latrines. After the spot checks, the team would go to the central point identified by the supervisor, where the supervisor would spin the pen to determine the directions in which the enumerators would enroll households. The supervisors checked on the enumerators to ensure they followed the sampling rules and also occasionally accompanied them into households to ensure they were asking the questions correctly and checked the data collection forms for missing values and contradictory answers.

### Ethics and consent.

Ethics approval was obtained from the London School of Hygiene and Tropical Medicine and the School of Medicine of the Kalinga Institute of Industrial Technology (KIIT) (India). Prior to enrollment, the enumerators read an information sheet describing the study, answered any questions, and asked for written consent to participate. The study participants received no compensation for their participation and were free to withdraw from the study at any time. Anonymity was ensured through the use of household identification numbers.

### Data entry and analysis.

Data were double entered using EpiData 3.1 (EpiData Association, Odense, Denmark) and analyzed using STATA version 14 (StataCorp). The description of child feces management behaviors was stratified according to the mobility category of the children. Child feces disposal was categorized as ‘safe’ if the child’s feces ended up in a latrine and ‘improved’ if the latrine was considered improved according to the JMP (flush/pour flush with pit/closed sewer and pit latrine with slab).^28^ The data used for describing the behaviors were from questions on the last time the child defecated, which was collected for each child under 5 years of age in the household. This was complemented with data collected at the household level including data on handwashing and latrine training, and demonstration data on whether the defecation place was cleaned (only collected through demonstrations), which was only asked once per household about the youngest child in the household if there were more than one child. Data about the last time the child defecated were used as this was collected for each child in the household and thus provided a larger data set. Descriptive measures in the form of frequencies and percentages or median, interquartile range, and minimum, maximum values were prepared to summarize the data presented in the tables and figure.

## RESULTS

A total of 694 households, with 852 children < 5 years of age, were enrolled from 42 slums. There was an average of 16.5 respondents per slum (range: 3–20). The primary caregiver of the youngest child in the household who was the respondent for the survey, was mostly the mother of the child (96.3%) (Table 1) and most were not engaged in income generation (self-reported as housewives, 90.9%, data not shown).

**Table 1:** Household characteristics

	*N*	%	Median (IQR)	Min-max
Demographics				
Gender of head of HH	694			
Male	567	81.7		
Female	127	18.3		
Number of persons per household	694		5 (3.0)	2–17
Caregiver’s relationship to youngest child	694			
Mother of the child	668	96.3		
Other (father, grandmother, aunt, sister)	26	3.7		
Religion	694			
Hindu	654	94.2		
Muslim	32	4.6		
Christian	8	1.2		
Age of caregiver	694		26 (6.0)	18–75
Education of caregiver	694			
Illiterate	55	7.9		
Literate without formal schooling	57	8.2		
Some/completed primary school	135	19.5		
Completed secondary school	350	50.4		
Any higher level of education	97	14.0		
Type of household construction*	694			
Pucca	495	71.3		
Semi-pucca	152	21.9		
Kuchha	47	6.8		
Own a BPL/ AYY card†	694			
Yes	179	25.8		
No	506	72.9		
DK	9	1.3		
Type of latrine‡	694			
Private	264	38.0		
Shared	183	26.4		
Communal	202	29.1		
Not using a latrine	45	6.5		
Water source location (98.8% improved)	693			
In dwelling	221	31.9		
In compound	135	19.5		
Outside compound	337	48.6		
Number of children < 5 years of age per household	852		1 (0.0)	1–4
Gender of child	852			
Male	418	49.1		
Female	434	50.9		
Age of children (months)	852			
0–11	155	18.2		
12–23	191	22.4		
24–35	162	19.0		
36–47	175	20.5		
48–59	169	19.8		

* Pucca = concrete walls, floors and roof, or corrugated roof; Kuccha = mud, dung, plastic, wood (nondurable materials); semi-pucca = mix of pucca and kuchha.

† BPL = below poverty line, AYY = Antyodaya (extreme poverty) ration cards.

‡ Of any type: improved/unimproved.

The latrines reported to be used by the household the majority of the time were mostly private latrines (of any type improved/ unimproved) (38.0%), followed by communal latrines (29.1%) and shared (26.4%). Forty-five households (6.5%) reported not using any sanitation facility, despite having access to communal latrines. Among the 40 communal latrines that the participating households reported using, three had separate latrines specifically for children (two with six “seats” and one with nine “seats”), of which only one latrine seemed to be used.

While 45.4% of caregivers had heard of potties (referred to as “plastic latrines”), only 7.6% owned one (53/694) and of those 86.8% (46/53) showed it at the time of visit; 47.3% (328/694) of caregivers reported that they or other members of the household sometimes purchased diapers, of those 90.5% (297/328) agreed that diapers were too expensive to be used daily and only in 2.4% (8/328) of those households was there a child observed to be wearing a diaper at the time of visit.

Caregivers reported that the median age to train their child to use a latrine was 3 years (interquartile range (IQR): 2.0, range: 1–14 years, five said never) with median age being lowest for users of private latrines (median: 3, IQR: 2.0, range: 1–8) and shared latrines (median: 3, IQR: 2.0, range; 1–10), followed by communal latrines (median: 4, IQR: 2.0, range; 1–14) and households where no one uses sanitation facilities (median: 5, IQR: 1.0, range; 2–8). Caregivers expected their child to use a latrine by themselves by the median age of 5 years (IQR: 2.0, range: 1–14 years, three said never), this again increased according to the household sanitation facilities (median for private and shared latrine users: 5, median for communal latrine users and households were no one uses latrines: 6).

Complete data on defecation behaviors were available for 851 children, of which 631 could walk (ambulatory) and 220 were preambulatory. Overall, 25.5% (95% CI: 22.7–28.5) of the 851 children’s feces were reported to end up in the latrine the last time they defecated; 20.3% (95% CI: 17.8–23.2) defecated directly into latrine while the others had feces deposited there after defecating elsewhere. Only 13.5% (95% CI: 11.4–16.0) ended up in improved latrines (improved disposal). No household reported burying their child’s feces.

The main defecation place for preambulatory children was on the ground inside the household (40.9%) followed by on the ground in the compound (27.3%), and the main disposal sites were the garbage (30.0%) and the canal or drain (25.0%) (Table 2). When the child defecated on the ground (in household, compound, latrine cubicle, *N* = 151), 62.3% was directly on the ground, 18.5% was on cloth, 15.2% on paper and 2.0% on polythene, and 2.0% on oil cloth. 34.6% of preambulatory children were reported to have their feces washed with water (13.2%) or with water and soap (21.4%), mostly in bathing areas that tend to be directly connected to the open drains, or at water sources (e.g. river, canal, near hand pumps, near wells or tube wells); however, we did not specifically collect data on the disposal of the contaminated water. Only 5% of the preambulatory children’s feces ended up in the latrine the last time they defecated.

**Table 2:** Frequency of feces disposal sites of preambulatory children by site of defecation and on what they defecated (*N* = 220)*

	Disposal site													
	Thrown in garbage†		Thrown into canal/drain		Washed with water + soap‡		Washed with water only		Thrown outside§		Put/rinsed into latrine		Total	
Defecation site	*n*	(%)	*n*	(%)	*n*	(%)	*n*	(%)	*n*	(%)	*n*	(%)	*n*	(%)
On ground inside household	26	(39.4)	29	(52.7)	17	(36.2)	8	(27.6)	7	(58.3)	3	(27.3)	90	(40.9)
Directly on ground	17	(25.8)	22	(40.0)	3	(6.4)	2	(6.9)	5	(41.7)	3	(27.3)	52	(23.6)
On cloth	1	(1.5)	1	(1.8)	14	(29.8)	5	(17.2)	2	(16.7)	0	(0.0)	23	(10.5)
On paper	5	(7.6)	6	(10.9)	0	(0.0)	0	(0.0)	0	(0.0)	0	(0.0)	11	(5.0)
On polythene/oilcloth	3	(4.5)	0	(0.0)	0	(0.0)	1	(3.4)	0	(0.0)	0	(0.0)	4	(1.8)
On ground in compound	28	(42.4)	18	(32.7)	5	(10.6)	5	(17.2)	3	(25.0)	1	(9.1)	60	(27.3)
Directly on ground	21	(31.8)	15	(27.3)	3	(6.4)	1	(3.4)	2	(16.7)	0	(0.0)	42	(19.1)
On cloth	0	(0.0)	0	(0.0)	1	(2.1)	4	(13.8)	0	(0.0)	0	(0.0)	5	(2.3)
On paper	6	(9.1)	3	(5.5)	0	(0.0)	0	(0.0)	1	(8.3)	1	(9.1)	11	(5.0)
On polythene/oilcloth	1	(1.5)	0	(0.0)	1	(2.1)	0	(0.0)	0	(0.0)	0	(0.0)	2	(0.9)
On bed	2	(3.0)	5	(9.1)	22	(46.8)	11	(37.9)	1	(8.3)	4	(36.4)	45	(20.5)
On cloth	2	(3.0)	3	(5.5)	21	(44.7)	11	(37.9)	1	(8.3)	4	(36.4)	42	(19.1)
On paper	0	(0.0)	0	(0.0)	1	(2.1)	0	(0.0)	0	(0.0)	0	(0.0)	1	(0.5)
On polythene/oilcloth	0	(0.0)	2	(3.6)	0	(0.0)	0	(0.0)	0	(0.0)	0	(0.0)	2	(0.9)
In cloth nappy/pants	1	(1.5)	1	(1.8)	3	(6.4)	5	(17.2)	0	(0.0)	1	(9.1)	11	(5.0)
In diaper	8	(12.1)	0	(0.0)	0	(0.0)	0	(0.0)	0	(0.0)	1	(9.1)	9	(4.1)
In potty	0	(0.0)	2	(3.6)	0	(0.0)	0	(0.0)	1	(8.3)	1	(9.1)	4	(1.8)
On ground in latrine cubicle	1	(1.5)	0	(0.0)	0	(0.0)	0	(0.0)	0	(0.0)	0	(0.0)	1	(0.5)
On paper	1	(1.5)	0	(0.0)	0	(0.0)	0	(0.0)	0	(0.0)	0	(0.0)	1	(0.5)
Total	66	(30.0)	55	(25.0)	47	(21.4)	29	(13.2)	12	(5.5)	11	(5.0)	220	(100.0)

* The table is organized descending from the main defecation and disposal sites.

† At house compound, at dump, in dustbin, sweeper van‡;includes dettol/ detergent§;open field, rail tracks, outside compound, pond, roadside.

For ambulatory children, 27.4% defecated into a latrine, of which 49.1% (85/173) were improved latrines; most defecated on the ground in the compound (28.5%) (Table 3). When the child defecated on the ground (in compound, in house, on path, in latrine cubicle, in bathroom, on the road side, river side, or field, *N* = 364), 75.3% was directly on the ground, 23.6% on paper and 0.6% on polythene, 0.3% on a plank and 0.3% on oil cloth. While 32.6% of ambulatory children’s feces were reported to end up in the latrine, only 7.2% (33/458) of defecation events that occurred elsewhere than the latrine the last time the child defecated resulted in feces being disposed in the latrine. Ambulatory children’s feces were also reported to be disposed of in garbage (25.0%) and the canal or drain (20.9%).

**Table 3:** Frequency of feces disposal sites of ambulatory children by site of defecation and on what they defecated (*N* = 631)*

	Disposal site															
	Put/rinsed into latrine		Thrown in garbage†		Thrown into canal/drain		Left in the open		Thrown outside‡		Washed with water only		Washed water +soap§		Total	
Defecation site	*n*	(%)	*n*	(%)	*n*	(%)	*n*	(%)	*N*	(%)	*n*	(%)	*n*	(%)	*n*	(%)
On ground in compound	7	(3.4)	70	(44.3)	80	(60.6)	1	(1.3)	21	(45.7)	1	(16.7)	0	(0.0)	180	(28.5)
Directly on ground	4	(1.9)	46	(29.1)	54	(40.9)	1	(1.3)	20	(43.5)	1	(16.7)	0	(0.0)	126	(20.0)
On paper	3	(1.5)	23	(14.6)	26	(19.7)	0	(0.0)	1	(2.2)	0	(0.0)	0	(0.0)	53	(8.4)
On polythene/oilcloth/plank	0	(0.0)	1	(0.6)	0	(0.0)	0	(0.0)	0	(0.0)	0	(0.0)	0	(0.0)	1	(0.2)
Directly into latrine	173	(84.0)	0	(0.0)	0	(0.0)	0	(0.0)	0	(0.0)	0	(0.0)	0	(0.0)	173	(27.4)
Side path	2	(1.0)	56	(35.4)	24	(18.2)	0	(0.0)	14	(30.4)	0	(0.0)	0	(0.0)	96	(15.2)
Directly on ground	1	(0.5)	43	(27.2)	20	(15.2)	0	(0.0)	12	(26.1)	0	(0.0)	0	(0.0)	76	(12.0)
On paper	1	(0.5)	12	(7.6)	4	(3.0)	0	(0.0)	1	(2.2)	0	(0.0)	0	(0.0)	18	(2.9)
On polythene/oilcloth/plank	0	(0.0)	1	(0.6)	0	(0.0)	0	(0.0)	1	(2.2)	0	(0.0)	0	(0.0)	2	(0.3)
In drain	0	(0.0)	0	(0.0)	0	(0.0)	68	(85.0)	0	(0.0)	0	(0.0)	0	(0.0)	68	(10.8)
On ground inside household	3	(1.5)	25	(15.8)	21	(15.9)	0	(0.0)	7	(15.2)	1	(16.7)	0	(0.0)	57	(9.0)
Directly on ground	3	(1.5)	18	(11.4)	15	(11.4)	0	(0.0)	4	(8.7)	1	(16.7)	0	(0.0)	41	(6.5)
On paper	0	(0.0)	7	(4.4)	5	(3.8)	0	(0.0)	3	(6.5)	0	(0.0)	0	(0.0)	15	(2.4)
On polythene/oilcloth/plank	0	(0.0)	0	(0.0)	1	(0.8)	0	(0.0)	0	(0.0)	0	(0.0)	0	(0.0)	1	(0.2)
In potty	7	(3.4)	4	(2.5)	3	(2.3)	0	(0.0)	4	(8.7)	1	(16.7)	1	(33.3)	20	(3.2)
On ground in latrine cubicle‖	13	(6.3)	1	(0.6)	0	(0.0)	0	(0.0)	0	(0.0)	0	(0.0)	0	(0.0)	14	(2.2)
Roadside/riverside/field‖	0	(0.0)	1	(0.6)	0	(0.0)	11	(13.8)	0	(0.0)	0	(0.0)	0	(0.0)	12	(1.9)
Bathroom‖	0	(0.0)	0	(0.0)	3	(2.3)	0	(0.0)	0	(0.0)	2	(33.3)	0	(0.0)	5	(0.8)
On bed	0	(0.0)	0	(0.0)	0	(0.0)	0	(0.0)	0	(0.0)	1	(16.7)	2	(66.7)	3	(0.5)
On cloth	0	(0.0)	0	(0.0)	0	(0.0)	0	(0.0)	0	(0.0)	1	(16.7)	2	(66.7)	3	(0.5)
In cloth nappy/pants	1	(0.5)	0	(0.0)	1	(0.8)	0	(0.0)	0	(0.0)	0	(0.0)	0	(0.0)	2	(0.3)
In diaper	0	(0.0)	1	(0.6)	0	(0.0)	0	(0.0)	0	(0.0)	0	(0.0)	0	(0.0)	1	(0.2)
Total	206	(32.6)	158	(25.0)	132	(20.9)	80	(12.7)	46	(7.3)	6	(1.0)	3	(0.5)	631	(100.0)

* The table is organized descending from the main defecation and disposal sites.

† At house compound, at dump, in dustbin, sweeper van‡;open field, rail tracks, outside compound, pond, roadside§;includes dettol/ detergent‖;all directly on ground.

The main tool used to pick up and dispose of the stools for preambulatory children was cloth (45.5%, 100/220), mostly after the child had defecated on it (67%, 67/100) followed by paper (37.7%, 83/220), mostly after the child had defecated on the ground (66.3%, 55/83). For ambulatory children, paper was the main disposal tool (68.8%, 260/378), either after the child had defecated on it (33.1%, 86/260) or just used to pick up after the child defecated on the ground (66.5%, 173/260) or oil cloth (0.4%, 1/260).

After defecation, caregivers reported washing their child’s bottom; however, this was mostly with water only (53.1% for preambulatory and 78.5% for ambulatory children). After disposing of child feces, 99.6% of caregivers (529/531) reported washing their hands, 69.9% (370/529) reported to have a specific place to wash their hands and in 62.2% (230/370) of households, soap and water was observed to be available at that place (100/150 preambulatory and 130/220 ambulatory).

**Figure 1.**
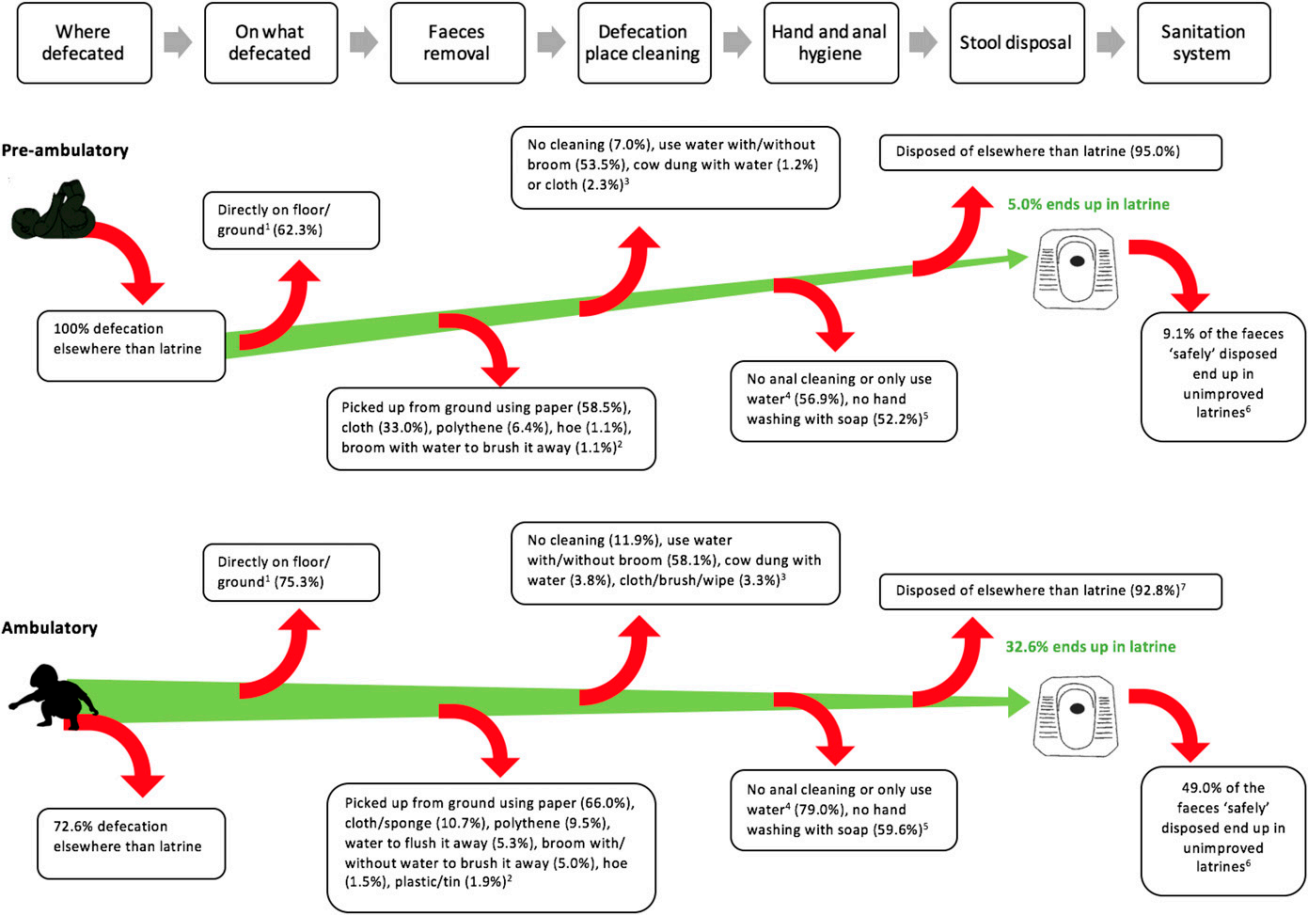
Child feces exposure pathway. 1—if child defecated on ground (*N* = 151 preambulatory and 364 ambulatory children), that is, on ground in latrine cubicle, on the roadside, on the path near the house, in the household, in the household compound, in a field, by the river, in the bathroom floor. 2—if child defecated elsewhere than latrine, potty, diaper, nappy, bed, drain and if the feces were not left in the open (i.e., not disposed of) and the child defecated directly on the ground (*N* = 94 preambulatory and 262 ambulatory children). 3—estimated using demonstration data for youngest child (total = 211 preambulatory [four missing] and 483 ambulatory children [three missing]), when the child was reported to defecate on the ground directly of the latrine cubicle, path near the house, in the household, in the household compound, bathroom (*N* = 86 preambulatory and 210 ambulatory). 4—does the caregiver wash the bottom of the child after defecation, using data on whether youngest child was ambulatory or not (only one response per household) (*N* = 211 preambulatory and 483 ambulatory children). For three ambulatory children, the caregiver said the child cleans his/her bottom by themselves so there are no data on those children. 5—based on caregivers not washing hands (only two preambulatory), not having a specific place to wash their hands or there being a handwashing facility but with no water and soap, if caregivers demonstrated/reported disposing of their children’s feces (i.e., the question was asked if the feces were not left in the open, or children did not directly defecate in the latrine) (*N* = 211 preambulatory and 324 ambulatory but data are missing for two preambulatory and two ambulatory children). 6—1/ 11 safely disposed feces of preambulatory feces end up in unimproved latrines, 101/ 206 safely disposed feces of ambulatory children end up in unimproved latrines. 7—if child defecated elsewhere than latrine (*N* = 458). This figure appears in color at www.ajtmh.org.

Our research shows other points during child feces management when fecal pathogens enter the environment causing the potential for exposure. Figure 1 illustrates these potential sources of exposure both for preambulatory and ambulatory children. First, the child may defecate on the ground directly as opposed to on paper or plastic. Indeed, of the defecation events on the ground, 62.3% were directly on the ground for preambulatory and 75.3% for ambulatory children. Second, the feces may not be picked up (12.7% for ambulatory children) or not be picked up efficiently leaving some pathogens at the defecation place. Third, the feces may be picked with a tool that may not efficiently prevent hand contamination, such as a cloth or paper. The ground may then not be cleaned with anything (7.0% for preambulatory and 11.9% for ambulatory children) or with water only (53.5% preambulatory and 58.1% ambulatory) or cow dung, creating the potential for adding pathogens on the floor.^38^ Finally, most caregivers did not have a specific place to wash their hands after disposing of their child’s feces (30.1%) or had a facility but there was no soap and water available at that place (26.5%), and most caregivers used only water for anal cleaning of their child after defecation (71.8%). Each of these represents a critical control point that simple monitoring of the place of disposal does not currently address.

## DISCUSSION

In this article, we describe defecation and feces disposal practices of children living in slums in Bhubaneswar and Cuttack. We attempted to describe the child feces management process to show the multiple pathways in which child feces may enter the environment.

Most of the feces ended up in the household waste and in open drains or canals. Disposal of child feces with garbage was considered neither safe nor improved in an expert consultation due to the proximity of the garbage to the domestic environment among other reasons.^39^ The defecation of children and the disposal of their stools in drains, may further contaminate the drains with fecal microbes, a possibly important source of exposure when children have contact with the drains.^40^

While we collected data from households that had access to a latrine (any type), we found that the majority of feces ended up in the environment and few were disposed of safely in a latrine, even fewer into an improved latrine. Safe disposal mostly took place where ambulatory children defecated directly in the latrine. On few occasions when the child defecated elsewhere than the latrine were the feces disposed of in the latrine. This is a finding similar to what we saw in rural areas of Odisha.^26,27^ Qualitative research in rural Odisha has described sanitation rituals that prohibit safe disposal in a latrine since a change or wash of clothes is prescribed after entering the latrine or stepping on the squatting pan to dispose of children’s feces.^41^

While 5% of preambulatory and 32.6% of ambulatory children’s feces were reported to end up in the latrine, even this may overstate the extent to which these feces are safely managed. Indeed, large amounts of feces that end up in the latrine are actually returned to the environment during leaks in the entire fecal sludge management chain (fecal waste flow diagram^25^).

In addition to the defecation and disposal elsewhere than the latrine, there are several points during the child feces management process that may create exposure to feces. This suggests that current monitoring of child feces—which is limited to the place of disposal—may not be adequate to address the risks presented by child feces. A “Child Feces Safety Plan,” modeled after the WHO’s water safety plans^42^ and recent sanitation safety planning^43^ may be helpful in highlighting the hazardous control points in the management of child feces.

Capturing all such potential sources of exposure would obviously complicate international monitoring. Further research may help quantify the risk of the different child feces management practices and thus identify key practices that may have the highest impact on health. There may be some practices that may present more protection from others in terms of contamination. For example, is using pants or cloth nappies more safe than the child defecating on the ground before being disposed, even if pants and cloth nappies may not be completely leak-proof? And how do cloth nappies compare with disposable diapers considering the diapers are often disposed of in the garbage whereas cloth nappies are mostly washed with the water ending up in the environment?

Moreover, practices vary by age, which is reflected in the differences in child feces management between preambulatory and ambulatory children. Younger preambulatory children may be the major priority for intervention since they are unable to use the latrines directly, few of them use potties/diapers, and they defecate closer to the domestic environment and mostly on the floor or cloth. Additionally, they spend more time in the household environment, thus potentially creating exposure for other household members, particularly children. Since children are taught how to use a latrine at about 3 years of age, the main gap is before that age.

## LIMITATIONS

There may be other behaviors that were not quantified in our research or were not captured accurately, for example, handwashing of the children after defecation, which has been found to be poor in rural Bangladesh.^24^ This aspect should be investigated for children being trained to use the toilet. We did not collect data on whether the tool used for child feces disposal/removal was cleaned afterwards, which is also a step of child feces management that may create a potential risk for exposure.^44^ What happens with the water when the main disposal was washed with water or with water and soap is unclear, but it is assumed to end up in the environment where the cloth/ nappy, etc. is washed. Future research should quantify where the water ends up, as well as the other feces management steps. While we collected data on the consistency of the feces the last time the child defecated this does not indicate whether the child was sick with diarrhea but it was used to understand whether there were differences in disposal when feces are more liquid. This is, however, an important research question as presumably diarrhea feces may pose a more significant threat as they may contain more pathogens, thus the disposal of diarrhea feces may be an important question to ask. The temporality of events was not captured in the questionnaire and it may be relevant to know how long child feces remain at the defecation place before being disposed of etc. How consistent the disposal behavior is, would also be interesting as this has not been found to be the case in other studies.^26,44^

This study was intended to explore fecal management practices and not to estimate the prevalence of those practices in a particular community. In addition, it has been found that participants overreport “desirable” behaviors of child feces disposal when data are collected using questionnaires compared with structured observations.^45,46^ We tried to minimize this by using questions about the last time children defecated^32^ and ask participants to demonstrate what they would do if their youngest child defecated at the time of visit. In addition, recent evidence suggests that reported and observed behavior were very similar.^24^
